# Prediction of the Outcome of Cochlear Implantation in the Patients with Congenital Cytomegalovirus Infection based on Magnetic Resonance Imaging Characteristics

**DOI:** 10.3390/jcm8020136

**Published:** 2019-01-24

**Authors:** Jae Joon Han, Yun Jung Bae, Seul Ki Song, Jae-Jin Song, Ja-Won Koo, Jun Ho Lee, Seung Ha Oh, Bong Jik Kim, Byung Yoon Choi

**Affiliations:** 1 Department of Otorhinolaryngology-Head and Neck Surgery, Soonchunhyang University College of Medicine, Seoul Hospital, Seoul 04401, Korea; seagulla@naver.com; 2 Department of Radiology, Seoul National University Bundang Hospital, Seongnam 13620, Korea; bae729@gmail.com; 3 Department of Otorhinolaryngology-Head and Neck Surgery, Seoul National University Bundang Hospital, Seongnam 13620, Korea; ssgi87@naver.com (S.K.S.); jjsong96@gmail.com (J.-J.S.); jwkoo99@snu.ac.kr (J.-W.K.); 4 Department of Otorhinolaryngology-Head and Neck Surgery, Seoul National University Hospital, Seoul 03080, Korea; junlee@snu.ac.kr (J.H.L.); shaoh@snu.ac.kr (S.H.O.); 5 Department of Otorhinolaryngology-Head and Neck Surgery, Chungnam National University, College of Medicine, Daejeon 35015, Korea

**Keywords:** cytomegalovirus, hearing loss, cochlear implantation, speech perception, outcome, magnetic resonance imaging, radiologic biomarker, white matter

## Abstract

The goal of this study was to elucidate radiologic biomarker that can predict the outcome of cochlear implantation (CI) in congenital cytomegalovirus (cCMV) related deafness. A retrospective survey of speech perception after CI and an evaluation of brain magnetic resonance imaging (MRI) findings were performed in 10 cochlear implantees with cCMV-related prelingual deafness. Specifically, a special attention was paid to the degree of white matter (WM) abnormality shown in brain MRI, which was used to divide our cohort into two groups: The mild and severe pathology groups. Age-matched prelingual deaf patients with idiopathic sensorineural hearing loss were selected as controls. Subjects in mild pathology groups showed higher a Category of Auditory Performance (CAP) score (5.2 ± 0.8) than those with severe pathologies (3.4 ± 1.5) (*P* = 0.041). Importantly, speech performance from subjects with mild pathology was comparable to that of the control group (mean CAP score of 5.2 ± 0.8 vs. 5.1 ± 1.2) (*P* = 0.898). Mild pathologies related to the limited WM lesion in MRI not accompanied by severe MRI pathologies, such as diffuse WM abnormality, myelination delay, ventriculomegaly, migration abnormality, and cerebellar hypoplasia, can be tolerated and do not adversely affect the CI outcome in cCMV deafness.

## 1. Introduction

Cytomegalovirus is the most common cause of congenital infection, with an overall birth prevalence of 0.64% [1]; congenital cytomegalovirus (cCMV) infection is believed to be an important etiology of sensorineural hearing loss (SNHL) in children, reaching up to 12% [2]. About 10% of infants with cCMV infection are symptomatic and exhibit any one of the following clinical findings: Petechiae, jaundice with hyperbilirubinemia (direct bilirubin > 2 mg/dL), hepatosplenomegaly, thrombocytopenia, microcephaly, chorioretinitis, and seizures [3]. About half of those with symptomatic cCMV infection end up developing either congenital (70%) or delayed-onset (30%) hearing loss [4]. On the other hand, the prevalence of SNHL is 7~15% in children with asymptomatic cCMV infection [3,5,6,7]. 

Cochlear implantation (CI) has been shown to be an effective auditory rehabilitation method for patients with severe-to-profound hearing loss. Children with cCMV infection have also been potential candidates for CI. The outcome of CI in cCMV patients remains controversial. A substantial portion of cCMV deafness manifests in an asymmetrical, progressive fashion with significant residual hearing [8]. In these cases, satisfactory speech development can be anticipated. However, to date, predicting the outcome of prelingual bilateral profound hearing loss due to cCMV infection remains infeasible. In a previous comparative studies, the cCMV patients showed comparable CI outcome to the groups with idiopathic SNHL [9,10] or SNHL by GJB2 mutation [11,12]. Conversely, some studies reported variable outcomes of CI [9,13,14]. After 4 years of CI use, 64% of children with CMV-related hearing loss were able to recognize open-set words [15], although postoperative speech perception was limited to the perception of sound in some patients. With long-term use of CI (about 10 years), auditory perceptive performance improved—with improvement in the ability to recognize open-set words—in most patients [16]. However, the ratio of language age to chronological age was variable from 13 to 63% [16]. 

To date, several parameters have been speculated to influence the outcome of CI in cCMV patients [10,11,14,15,16,17]. The variable outcome of CI might be attributed to the degree, onset, progression, and duration of hearing loss, as well as the developmental delay and motor or cognitive disability. Recently, brain abnormalities on MRI were shown to be related to the insufficient progress of speech performance after CI in cCMV patients [12,18], although these previous studies failed to correlate the characteristic findings of MRI with the CI outcome [19]. Given this, we aimed to further evaluate brain MRI findings as the potential prognostic factors predicting the degree of speech development after CI in patients with documented prelingual cCMV-related bilateral deafness. In addition, we tried to provide a realistic expectation after CI for children with suspected viral etiology based on brain MRI findings.

## 2. Materials and Methods

### 2.1. Participants

Between September 2009 and September 2016, 10 patients (8 males, 2 females), who were diagnosed with cCMV infection with severe-to-profound hearing loss and underwent CI at Seoul National University Hospital and Seoul National University Bundang Hospital, were included (Table 1). The diagnosis of cCMV infection was confirmed when one of the following criteria was satisfied: (1) The child was found to have CMV in the urine samples with a culture or PCR test within 2 weeks of birth; or (2) the child was positive for anti-CMV IgM titers in a serum collected at or shortly after birth. Among 10 participants, criteria 1 using urine sample was satisfied in four patients (CH-3, 4, 5, 9). The other six patients were diagnosed as cCMV infection using anti-CMV IgM titers in a serum (criteria 2) (CH-1, 2, 6, 7, 8, 10). Diagnostic work-up for cCMV infection was performed when the symptoms or signs related to cCMV infection were presented at birth, such as neonatal jaundice, stay at neonatal intensive care unit, preterm birth, or intrauterine growth retardation. Three patients received antiviral therapy by intravenous Ganciclovir or oral Valganciclovir, or both for six weeks (CH-2, 3, 9). None of side effects such as bone marrow suppression was present. All of them showed no change of hearing level despite of antiviral therapy, and CI was performed. The age at operation, age of deafness onset, deafness duration, speech perception ability, and image findings of MRI were retrospectively reviewed. The mean age at operation of the 10 patients was 2.2 ± 1.6 (1.1–6.4) years. Most of the participants were referred with abnormal results from universal newborn hearing screening (UNHS), except one patient (CH-4) who passed the UNHS, but later developed bilateral deafness at age 2 years. All patients showed bilateral, severe-to-profound prelingual SNHL. The cCMV cases retaining significant residual hearing—either unilaterally or bilaterally until the peri-lingual period—were excluded.

Four experienced surgeons, including the authors of this study (J.L., S.O. and B.C.), performed the CI without any postoperative complications. Among the 10 participants, six patients (CH-2, 5, 7, 8, 9, 10) underwent a second CI surgery, including three cases of simultaneous bilateral CI (CH-7, 8, 9), and the average age of the second CI placement was 3.6 ± 4.4 years (ranging between 1.1 and 12.4 years).

Auditory rehabilitation using hearing aids and CI was applied in all of the participants preoperatively and postoperatively. Every participant tried bilateral hearing aids at least 3 months before CI. After surgery, consistent speech therapy was performed during the first 3 years after implantation. At least two sessions of speech therapy per a week were applied in all participants, and the patients were encouraged consistently to use the CI device for over 8 h per day.

To perform a comparative evaluation of the CI outcome in patients with cCMV infection, we allocated 10 patients who had congenital prelingual severe-to-profound hearing loss but without inner ear anomalies and underwent CI as a control group. All of the patients in the control group had idiopathic SNHL, and their age of operation was matched with that of cCMV patients. This study was approved by the institutional review board of Seoul National University Bundang Hospital (No. B-1612/376-106) and Seoul National University Hospital (No. J-1704-109-847) for research involving human subjects. All procedures were performed in accordance with the relevant guidelines and regulations. Written informed consent was obtained from the parents or legal guardians of all participants. All data generated or analyzed during this study are included in this published article.

### 2.2. Audiological Evaluation

Postoperative speech perception performance of the participants was evaluated using the Category of Auditory Performance (CAP) score. The CAP score ranges from 0 (no awareness of environmental sound) to 7 (use of telephone with known speaker), denoting the degree of speech perception performance [20]. The speech evaluation was performed repeatedly until 3 years after CI (3-, 6-, 12-, 18-, 24-, 36 months). 

### 2.3. Magnetic Resonance Imaging (MRI)

The axial and sagittal spin-echo (SE) T1-weighted images and the axial and/or coronal fast SE T2-weighted images were taken using 1.5 T scanners in all patients. In some cases, an inversion recovery T1-weighted image, T2*-gradient recalled-echo image, fluid attenuated inversion recovery (FLAIR) image, and heavily T2-weighted image were obtained for a more precise evaluation of myelin abnormalities, parenchymal pathologies, ventricles, nerves in the internal auditory canal, or cochlea. 

To assess the association between MR image findings and postoperative speech perception, a detailed image review was performed by a neuro-radiologist with extensive experience (over 10 years) who was blind to speech performance after CI. The typical MRI findings of cCMV infection include cerebral white matter (WM) abnormalities of variable extents, with or without cerebral gyral abnormalities [21]. In detail, the WM abnormalities on MRI—i.e., high signal intensities on the T2-weighted image (T2WI)—were evaluated according to the extent (none, absent T2-high signal foci; multifocal, multiple punctate T2-high signal foci; extensive, beginning confluence of the T2-high signal lesions; and diffuse, large confluence of the T2-high signal lesions or diffuse involvement of the entire WM) and the involvement of arcuate fiber, periventricular, or deep WM. The distribution of WM abnormality was evaluated according to the brain region—frontal, parietal, occipital, and temporal lobes [21]. Additionally, myelination delay was also evaluated. Other MRI findings related to cCMV infection, such as hippocampal dysplasia, cerebellar hypoplasia, and migration disorder, were also reviewed [22,23,24]. Subjects with any of the following findings on brain MR images were included in the ‘severe pathology group’: Diffuse WM abnormality, myelination delay, ventriculomegaly, cerebellar hypoplasia, or migration disorder. The mild pathology group, on the other hand, included those without any severe pathology. 

### 2.4. Statistical Analysis

All results are presented as the means ± standard deviations. Statistical analyses were performed using SPSS software (ver. 18.0; SPSS, Chicago, IL, USA). *P* values < 0.05 were considered to indicate statistical significance. The comparative analyses of postoperative CAP score according to the presence or absence of cCMV-related MRI findings of brain were performed with Mann-Whitney U-test.

## 3. Results

### 3.1. Speech Perception after CI

A total of 10 participants showed significant improvement in speech perception performance after CI; the preoperative average CAP score was 0.3 ± 0.7, and the 24-month postoperative average CAP score was 4.3 ± 1.5 (Table 1). Among them, three patients (CH-1, 3, 5) showed global developmental delay preoperatively, and the delay persisted even after CI. They showed poor CI outcome after CI (CAP score at 2 years after CI, 3.0 ± 1.7) than the others who did not show such a developmental delay (4.9 ± 1.1); however, this was without statistical significance (*P* = 0.079).

### 3.2. MRI Findings and Its Correlation with CI Outcome

**Figure 1:**
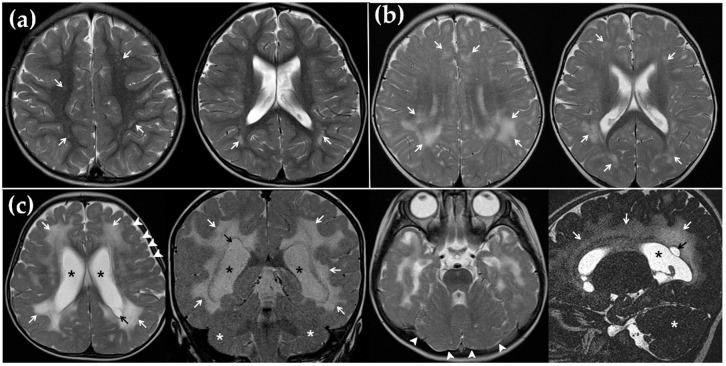
Various features of T2-weighted MR images in patients with cytomegalovirus infection. (**a**) Multifocal white matter (WM) lesions (white arrows) in deep WM of frontal and parietal lobe (CH-4). (**b**) Extensive WM lesions (white arrows) in periventricular and deep WM of whole brain areas (CH-9). (**c**) Diffuse WM lesion (white arrows) combined with periventricular cyst (black arrow), ventriculomegaly (black asterisk), cerebellar hypoplasia (white asterisk), and polymicrogyria (white arrowhead) (CH-3).

Among the 10 cochlear implantees due to cCMV deafness, only one patient (CH-10) (10%) showed no noticeable abnormality on MR images. Interestingly, half of the subjects had ventriculomegaly (Figure 1c) and showed significantly lower CAP scores at 2 years after CI than those with normal sized ventricles (3.4 ± 1.5, 5.2 ± 0.8, respectively; *P* = 0.041) (Table 2). Additionally, worse CI outcomes were noted in subjects with myelination delay on image findings (*n* = 2, 2.5 ± 2.1) (Figure 1c) than those without (*n* = 8, 4.8 ± 1.0), albeit without statistical significance due to the small sample size (Table 2). Furthermore, as WM abnormalities became extensive, the 2-year postoperative CAP score tended to decrease; however, statistical analysis was not feasible due to the small sample size. The group with normal WM (*n* = 1, CH-10), multifocal (*n* = 3, CH-4, 6, 8) (Figure 1a), extensive (*n* = 4, CH-2, 5, 7, 9) (Figure 1b), and diffuse (*n* = 2, CH-1, 3) (Figure 1c) WM lesions showed a CAP score of 6, 4.7 ± 0.6, 4.5 ± 1.3, and 4.0, respectively (Table 1). The image finding of cerebellar hypoplasia (*n* = 2) (Figure 1c) also merited attention: The 2-year postoperative CAP score of the two subjects (2.5 ± 2.1) seemed to be lower than that of those (4.8 ± 1.0) without cerebellar hypoplasia, despite the lack of statistical significance due to the small sample size (*P* = 0.107) (Table 2). Two patients with migration disorder also tended to show poorer CI outcomes than those without it (3.5 ± 0.7, 4.5 ± 1.6, *P* = 0.179) (Figure 1c). None of the subjects in our cohort showed hippocampal dysplasia. 

We further detailed the localization of WM abnormalities in the brain (Table 1). Seven patients showed WM abnormality in the periventricular area (*n* = 7/9, 78%); however, the 2-year postoperative CAP score was not significantly different between the groups with and without periventricular involvement (4.6 ± 1.0 and 5.0 ± 1.4, respectively; *P* = 0.648) (Table 2). There was also no difference in the 2-year postoperative CAP score between the groups with and without involvement of deep WM, arcuate fiber, the frontal lobe, the parietal lobe, the occipital lobe, and the temporal lobe (Table 2). 

**Figure 2:**
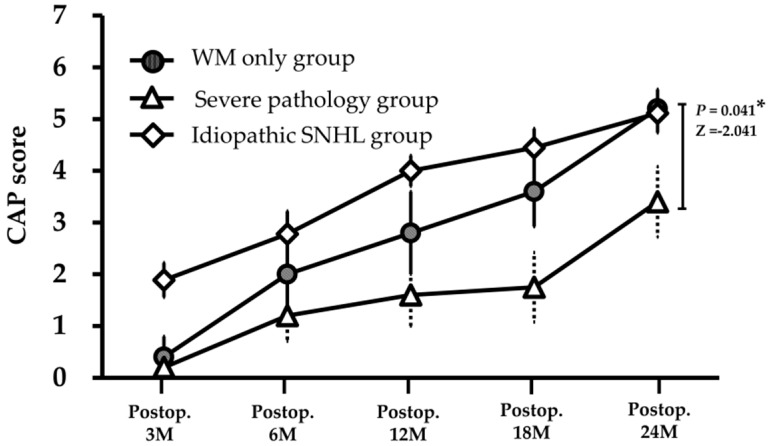
The progression of speech perception performance after cochlear implantation (CI) in patients with congenital cytomegalovirus (cCMV) infection and idiopathic sensorineural hearing loss (SNHL). Among the participants with cCMV infection, the mild pathology group having only limited white matter (WM) abnormalities in their MRIs showed rapid progression of speech perception performance after CI. At 2 years after CI, the categories of auditory performance score of mild pathology group was similar to that of the idiopathic SNHL group, whereas the CI outcomes of the severe pathology group with one or more of cCMV infection-related severe pathologic MRI findings, was significantly poorer than the mild pathology group and idiopathic SNHL group.

When we analyzed the CI outcomes depending on the presence of severe pathology on brain MRI, the mild pathology group (CMV-4, 6, 7, 9, 10) without any severe pathology in the brain showed a better CI outcome (mean CAP score of 5.2 ± 0.8) than the severe pathology group (3.4 ± 1.5) (*P* = 0.041) (Figure 2 and Table 1). The speech performance exclusively from the mild pathology group rapidly improved after CI, reaching a similar level to that obtained by the control group at 2 years after CI (CAP, 5.1 ± 1.2) (*P* = 0.898). On the other hand, the CI outcome of the severe pathology group (CH-1, 2, 3, 5, 8) was significantly worse than that of the idiopathic SNHL group (CAP, 3.4 ± 1.5 vs. 5.1 ± 1.2) (*P* = 0.045) (Figure 2).

## 4. Discussion

Although CI is known to be effective for patients with cCMV-related hearing loss, the outcome remains variable from person to person [15,16]. In the present study, we showed that patients with a normal or partial WM abnormality on MRI showed good speech perception performance after CI, at least comparable to the performance obtained by idiopathic SNHL patients. 

WM is located underneath the gray matter in the brain and is composed of neuronal fibers and myelin that covers the WM. Autoimmune diseases, such as Guillain-Barre syndrome or multiple sclerosis, inherited diseases, such as Charcot-Marie-Tooth disease, and external damages, including infection, trauma, toxins, or asphyxia, could cause structural changes in the myelin and WM [25]. Normally, myelinated WM shows a higher signal on T1-wieghted images (T1WI) and a lower signal on T2WI than gray matter. If there is a problem with the myelination of WM, the signal intensity of WM becomes higher than that of gray matter on T2WI (Figure 1) [26]. While the elevated signal intensity of WM on T2WI was consistently evident and greatly manifested in the myelination disorder, the T1 signal of cerebral WM in such cases could vary according to the degree of myelination and age [26].

Because myelin regulates the conduction velocity of neuronal fiber and synaptic plasticity, WM abnormalities are likely to be closely correlated with cognition, processing, and neurodevelopment [25]. When WM abnormalities—including WM volume loss, ventriculomegaly, cysts, WM signal abnormalities, and delayed myelination—are discovered in very preterm infants, the prognosis of neurodevelopment and cognitive functioning seems poor [27,28,29]. The degree of WM abnormalities could be evaluated with various rating systems using MR images [30], and the severity and extensiveness of WM abnormalities are reported to be correlated with cognitive and motor delay as well as neurosensory impairment in preterm infants [29]. A postnatal prognosis of SNHL was also related to the degree of WM abnormality [22]. Therefore, it may be safe to assert that WM abnormalities in cCMV patients might adversely affect speech perception and processing after CI. 

In our participants, two patients (CH-1, 3) showed a diffuse distribution of WM abnormality combined with myelination delay, ventriculomegaly, and cerebellar dysplasia (Figure 1c and Table 1). As expected with these severe WM abnormalities, the two patients had pervasive developmental delay, and accordingly their speech perception performance was limited in the level of simple sound perception (CAP 1) or discrimination of simple speech sound (CAP 4) at 2 years after CI (Table 1). Therefore, diffuse WM abnormality with myelination delay, ventriculomegaly, or cerebellar dysplasia on MRI images might be related to poor outcomes of CI in the patients with cCMV infection. In contrast, the mild pathology group (CMV-4, 6, 7, 9, 10) without severe pathology on MRI (Figure 1a,b) in our present study showed a good outcome and even comparable CAP scores with the idiopathic SNHL group (Figure 2). Therefore, outcomes of CI with pathologies related to the limited WM lesions not accompanied by such severe pathologies appear satisfactory.

In addition to WM abnormalities, cCMV infection of the brain could also be correlated with migration disorder and cerebellar hypoplasia (Figure 1c). Cortical dysplasia with migration disorder, such as lissencephaly or pachy/polymicrogyria, was known to be related to severe psychomotor retardation, cerebral palsy, and epilepsy [31,32]. Considering the relationship between cognitive skills, auditory processing, and language development, cortical dysplasia may be associated with poor prognosis after CI [17,33]. The MRI findings in the severe pathology group, including myelination delay, ventriculomegaly, migration abnormalities, and cerebellar hypoplasia, reflected a severe defect with respect to central nervous system (CNS) development and maturation, and the electrical stimulation via CI might be not beneficial in these cases (Figure 1 and Table 2).

Although the average speech performance after CI in patients with severe pathologic MR findings was significantly inferior overall to that in cCMV patients without such findings, a subset of these subjects with pathologic MR findings could still achieve improved speech perception and language performance with continuous auditory rehabilitation with CI. Three patients (CH-3, 5, 8) from the severe pathology group in this present study were able to achieve improved speech perception to the degree of being able to discriminate simple speech sounds (CAP 4) or understand common phrases without lip reading (CAP 5) via auditory rehabilitation with CI for 2 years (Figure 1c and Table 1). In accordance with this, a previous study reported a patient with bilateral polymicrogyria who achieved a level of telephone usage with a familiar person at 7 years after CI [19]. 

The differential CI outcomes between the non-diffuse WM pathology group and severe pathology group could be explained with the distinctive pathophysiology of cCMV-related hearing loss. First, the destruction of the cochlea and labyrinthitis were known to be the main pathophysiology of hearing loss in cCMV infection [34,35]. Cytomegalovirus and its DNA were detected in the perilymph of the cochlea in patients with cCMV infection [36,37], and the stria vascularis and outer hair cell were the primarily damaged structures in the cochlea [38,39]. Hearing impairment in cCMV infection could also result from damaged chromosomes [40,41], in which several genes related to the auditory neuropathy spectrum disorder (ANSD) could reside. In this case, the CI outcome would rely on whether the main lesion of the affected ANSD gene is postsynaptic. This situation could be suspected, when the mild pathologic group happens to be associated with a poor CI outcome. Lastly, cCMV infection in early gestational period could induce the defect of the CNS by disturbing the proliferation and differentiation of the neural progenitor cells in the CNS [42,43]. Therefore, hearing loss by cCMV might be attributed not only to the pathology of the cochlea, but also to a lesion beyond the cochlea, and finally even to an abnormality of the CNS. If there are obvious cCMV-related pathologic changes in the CNS, and if the neural network for speech perception and processes are affected, as in the severe pathology group, speech perception performance after CI would be poor overall, despite the presence of significant variability of CI outcomes in patients with cCMV infection. Currently, there are no radiological or clinical parameters that can predict the prognosis in those with severe pathology. On the other hand, if the involvement of cytomegalovirus is limited to within the presynaptic cochlear structures, and the CNS abnormality is absent or tolerable, as in the mild pathology group, with limited WM lesions, the auditory rehabilitation with CI would be satisfactory. In this situation, CI can directly stimulate the intact cochlear nerve and further deliver the signal to the upstream auditory pathways and ultimately to the CNS with only tolerable defects, detouring the cochlea, which is the main focus of cCMV-related pathology.

This study had several limitations. First, the number of patients was not sufficiently large to draw a solid conclusion about the negative predictive values of MRI findings on CI outcome, such as diffuse WM abnormality, myelination delay, ventriculomegaly, migration abnormality, and cerebellar hypoplasia. The patients who had those severe pathologies showed poor speech perception performance after CI, whereas the statistical significance was not evident due to the limited numbers in our cohort (Table 2). Second, well-known factors related to CI outcomes such as deafness duration, age at operation, residual hearing level, and interval to second CI, were not well controlled in our cohort due to the limited number of participants. For example, patient CH-10 underwent CI at age of 6 because her parents were reluctant to undergo CI and language development with hearing aids had appeared partially (preoperative CAP score was 2). In contrast, CI in patient CH-1 was delayed until the age of 3 due to severe comorbidities. If the neonate had no suspected symptoms for cCMV infection, diagnostic work-up for cCMV infection has not been tested generally, so the number of confirmed cCMV-infected CI implantees was limited. Recently, cCMV infection was proposed one of the most important etiologies of hearing loss, and the importance of diagnostic test for cCMV infection at birth was widely accepted worldwide. In the future, a well-designed study with a larger cohort would be possible, and MRI image findings with negative predictive value on CI outcomes would be clearly identified.

## 5. Conclusions

We identified tolerable CNS findings on MRI images that may indicate a good CI outcome in cCMV deafness. The pathologies related to the limited WM lesions not accompanied by diffuse WM abnormalities, myelination delay, ventriculomegaly, migration abnormalities, or cerebellar hypoplasia usually do not adversely impact CI outcomes. Our results could help clinicians to tease out a subset of cCMV deaf children with a good prognosis after CI, that may even be comparable to the control idiopathic SNHL group. This would be highly beneficial when counseling these families. However, severe pathologies on MRI that are significantly associated with poor overall CI outcomes do not necessarily lead to poor individual outcomes, thus mandating the emphasis on intensive auditory rehabilitation even in these cases.

## Figures and Tables

**Table 1:** MRI findings detected in patients with congenital cytomegalovirus infection and the outcome of cochlear implantation.

Patient ID	Sex	UNHS Rt/Lt	Hearing Thresholds † Rt/Lt (dB)	Age at 1st CI (year)	Age at 2nd CI (year)	Deaf Duration (years)	DD	CAP Score					White Matter Involvement								Myelination Delay	Ventriculomegaly	Hippocampal Dysplasia	Cerebellar Hypoplasia	Migration Disorder
								Pre	0.5	1	2	3	Distribution	Periventricular	Deep WM	Arcuate Fiber	Frontal Lobe	Parietal Lobe	Occipital Lobe	Temporal Lobe					
Severe pathology group																									
CH-1	M	R/R	85/90	3.0		3.0	●	0	1	1	1	1	Df	●	-	-	-	●	-	-	●	●	-	●	-
CH-2	M	R/R	115/110	1.2	2.4	1.2	-	0	1	2	3		Ex	●	●	●	●	●	-	●	-	●	-	-	●
CH-3	F	R/R	100/100	1.8		1.8	●	0	1	1	4	4	Df	●	●	●	●	●	●	●	●	●	-	●	●
CH-5	M	R/R	115/115	1.2	2.3	1.2	●	0	1	1	4	4	Ex	●	●	-	●	●	-	-	-	●	-	-	-
CH-8	M	R/R	100/110	1.3	1.3	1.3	-	1	4	4	5		Mf	●	●	-	●	●	●	●	-	●	-	-	-
Mild pathology group																									
CH-4	M	P/P	100/100	2.6		0.6	-	0	0	1	4		Mf	-	●	-	●	●	-	-	-	-	-	-	-
CH-6	M	R/R	100/110	1.2		1.2	-	0	2	4	5	6	Mf	●	-	-	-	●	-	-	-	-	-	-	-
CH-7	M	R/R	85/100	2.0	2.0	2.0.	-	0	4	5	5		Ex	●	●	●	●	●	●	●	-	-	-	-	-
CH-9	M	R/R	100/100	1.1	1.1	1.1	-	0	4	4	6	7	Ex	●	●	●	●	●	●	●	-	-	-	-	-
CH-10	F	R/R	95/105	6.4	12.4	6.4	-	2	4	4	6		none	-	-	-	-	-	-	-	-	-	-	-	-

M, male; F, female; Rt, right; Lt, left; UNHS, universal newborn hearing screening; R, refer; P, pass; CI, cochlear implantation; DD, developmental delay; Pre, preoperative; CAP, Categories of Auditory Performance; Mf, multifocal; Ex, extensive; Df, diffuse; WM, white matter; ● present, - not present; †, hearing thresholds measured with behavioral audiometry shortly before CI.

**Table 2:** The outcome comparison of cochlear implantation according to the findings of MRI abnormalities.

Abnormal Findings	Involved Area	Ratio of Positive Finding	Positive (CAP Score)	Negative (CAP Score)	*Z*	*P*
Involvement of WM abnormality	Periventricular	7/9 (78%)	4.6 ± 1.0	5.0 ± 1.4	−0.457	0.648
	Deep WM	7/9 (78%)	4.4 ± 1.0	5.5 ± 0.7	−1.370	0.171
	Arcuate fiber	4/9 (44%)	4.5 ± 1.3	4.8 ± 0.8	−0.382	0.702
	Frontal lobe	7/9 (78%)	4.4 ± 1.0	5.5 ± 0.7	−1.370	0.171
	Parietal lobe	8/9 (89%)	4.5 ± 0.9	6.0	−1.409	0.159
	Occipital lobe	4/9 (44%)	5.0 ± 0.8	4.4 ± 1.1	−0.891	0.373
	Temporal lobe	5/9 (56%)	4.6 ± 1.1	4.8 ± 1.0	−0.127	0.899
Myelination delay		2/10 (20%)	2.5 ± 2.1	4.8 ± 1.0	−1.611	0.107
Ventriculomegaly		5/10 (50%)	3.4 ± 1.5	5.2 ± 0.8	−2.041	0.041 *
Cerebellar hypoplasia		2/10 (20%)	2.5 ± 2.1	4.8 ± 1.0	−1.611	0.107
Migration disorder		2/10 (20%)	3.5 ± 0.7	4.5 ± 1.6	−1.343	0.179

WM, white matter; CAP, Categories of auditory performance; * *p* < 0.05.
